# pH-Dependent Water Clusters in Photoacid Solution: Real-Time Observation by ToF-SIMS at a Submicropore Confined Liquid-Vacuum Interface

**DOI:** 10.3389/fchem.2020.00731

**Published:** 2020-08-21

**Authors:** Ying-Ya Liu, Xin Hua, Zhiwei Zhang, Junji Zhang, Shaoze Zhang, Ping Hu, Yi-Tao Long

**Affiliations:** ^1^School of Chemistry and Molecular Engineering, East China University of Science and Technology, Shanghai, China; ^2^National Engineering Laboratory for Vacuum Metallurgy, Kunming University of Science and Technology, Kunming, China; ^3^Engineering Laboratory for Advanced Battery and Materials of Yunnan Province, Kunming University of Science and Technology, Kunming, China; ^4^State Key Laboratory of Analytical Chemistry for Life Science, School of Chemistry and Chemical Engineering, Nanjing University, Nanjing, China

**Keywords:** *in-situ* liquid ToF-SIMS, liquid-vacuum interface, photoacid, water cluster, hydrogen-bonding network

## Abstract

Water clusters are ubiquitously formed in aqueous solutions by hydrogen bonding, which is quite sensitive to various environment factors such as temperature, pressure, electrolytes, and pH. Investigation of how the environment has impact on water structure is important for further understanding of the nature of water and the interactions between water and solutes. In this work, pH-dependent water structure changes were studied by monitoring the changes for the size distribution of protonated water clusters by *in-situ* liquid ToF-SIMS. In combination with a light illumination system, *in-situ* liquid ToF-SIMS was used to real-time measure the changes of a light-activated organic photoacid under different light illumination conditions. Thus, the proton transfer and pH-mediated water cluster changes were analyzed in real-time. It was found that higher concentration of free protons could lead to a strengthened local hydrogen bonding network as well as relatively larger protonated water clusters in both organic acid and inorganic acid. Besides, the accumulation of protons at the liquid-vacuum interface under light illumination was observed owing to the affinity of organic molecules to the low-pressure gas phase. The application of *in-situ* liquid ToF-SIMS analysis in combination with *in-situ* light illumination system opened up an avenue to real-time investigate light-activated reactions. Besides, the results regarding water structure changes in acidic solutions showed important insights in related atmospheric and physiochemical processes.

## Introduction

Proton transfer (PT) along hydrogen-bond (HB) network in water plays a key role in multiple physical, chemical, and biological processes, such as acid-base reactions (Mohammed et al., 2005), organic synthesis (Guo et al., 2016), electro-catalysis (Badalyan and Stahl, 2016), and biological redox processes (Di Luca et al., 2017). According to the Grotthuss mechanism, protons transfer in the form of hydronium (H_3_O^+^) or hydroxide (OH^−^) by forming HBs with adjacent water molecules in aqueous solutions, resulting in the formation of protonated or hydroxide water clusters with different size and structure (Agmon, 1995). This mechanism explained the anomalously high mobility of hydronium than other ions (Miyazaki et al., 2004; Headrick et al., 2005; Natarajan et al., 2015; Chen et al., 2018; Daldrop et al., 2018). The ultrafast proton transfer *via* HB network is the basic of biological processes (Garczarek and Gerwert, 2006; Park et al., 2012). Water cluster is the dominant species in most biological systems and its size, structure, and structural transformation determine the dynamics of biological protons (Mohammed et al., 2007). Additionally, the protonated water cluster as a specific acid with unique proton transfer properties have a significant impact on catalysis. Thus, for better understanding of proton transfer processes in water, it is necessary to investigate the properties of water cluster in different environments (Ellmer et al., 2014; Gould et al., 2020). It was known that the structure of water clusters are sensitive to the environment, such as confined space, interface, temperature, pressure, electrolytes, and pH (Burnham et al., 2006; Li and Lazaridis, 2006; Wang et al., 2008). Thus, identifying the environmental effects on the dynamic and structural properties of water clusters at molecular level is of significant importance for better understanding the nature of water and the interactions between water and solutes.

Much attention has been paid to study how the temperature and electrolytes have effects on the dynamics of HB network and water cluster structure (Stirnemann et al., 2013; Zhao et al., 2015). However, the study of acid effect on HB network and water cluster structures, which is fundamental to some important chemical and physicochemical processes in the atmosphere and biological systems, are rare (Gutberlet et al., 2009). Investigation of PT process in acid solutions seemed more complicated due to the strong ability of acid to transfer protons to water and this process is largely solvation-dependent. So far, most researches regarding acid-water cluster interactions focused on the water cluster-assisted acid dissociation (Lee et al., 1996; Ding et al., 2003; Gutberlet et al., 2009; Li et al., 2019). But how the dissociated protons from acid affect the protonated water cluster structures is hardly known. Besides, most previous studies investigated the interactions between water clusters and inorganic acids, such as HCl, H_2_SO_4_, HF, and H_2_S. Nevertheless, the interaction between water clusters and organic acids, which is of significant importance for some key physiological processes, is little reported owing to the complexity of organic molecular structures.

Photoacids are a class of proton-containing organic compounds that undergo photo-induced proton dissociation and thermal reassociation (Johns et al., 2014). Upon irradiation, the acidity of the solution could become stronger, even to the degree of strong acids (Shi et al., 2011). Once the light is turned off, the conjugate base would be protonated to regenerate photoacids, showing excellent reversibility of the proton transfer process. Thus, photoacids could be used to noninvasively control the acidity of various reaction systems, such as dynamic self-assembly of nanoparticles, and photo polymerization (Fu et al., 2016; Liao, 2017; Yucknovsky et al., 2019). More importantly, the accurate control of solution acidity makes photoacids a good candidate for real-time and *in-situ* experimental study of proton transfer and the interaction between water and protons.

For decades, extensive experimental and computational studies have been used to investigate the water structure dynamics and the PT mechanism in aqueous solutions, including NMR spectroscopy (Meiboom, 1961), dielectric spectroscopy (Marcus and Hefter, 2006), ultrafast vibrational dynamic spectroscopies (Park and Fayer, 2007; Shalit et al., 2017), Femtosecond elastic second harmonic scattering (fs-ESHS) (Chen et al., 2016), X-ray diffraction (XRD) (Bouazizi et al., 2006), X-ray absorption spectroscopy (XAS) (Waluyo et al., 2014), surface-sensitive Sum Frequency Generation (SFG) (Ye et al., 2001), density functional theory (DFT) (Shi et al., 2017), and molecular dynamics (MD) simulations (Chen et al., 2018). However, the results from different methods sometimes resulted in diverse interpretations. This might be attributed to the local sensitivity of these techniques to specific properties of HB networks, such as the relaxation or reorientation time of water, the intramolecular O-H stretch vibration and HB dynamics, which greatly limited the understanding of proton-water interactions from molecular level. Mass spectrometry is a powerful method for the protonated water cluster studies which can be used to monitor the water cluster size distribution under various conditions. Time-of-flight secondary ion mass spectrometry (ToF-SIMS) is a highly surface sensitive technique with high spatial and time resolutions. In combination with a microfluidic reactor, *in-situ* liquid ToF-SIMS analysis could be conducted to overcome the limitation of liquid sample analysis in high vacuum environment of ToF-SIMS and thus provide important spatial and temporal chemical information at the pore-confined liquid-vacuum interface (Yang et al., 2011; Liu et al., 2018, 2019).

In this work, *in-situ* liquid ToF-SIMS in combination with a light illumination system was employed to real-time monitor the proton transfer between a light-triggered photoacid and water. The dynamics of the photoacid dissociation was measured. Besides, the effect of dissociated protons from photoacid on the dynamic of HB network and water cluster structure was detected in real-time. By comparing the size distribution of organic photoacid and inorganic acid solutions, the accumulation of organic photoacid molecules at the pore-confined liquid-vacuum interface was observed and interpreted.

## Materials and Methods

### Materials

Pure water (18.2 MΩ cm at 25°C) was from a Milli-Q purification system (Billerica, MA). Alcohol (anhydrous, ≥99%), sodium chloride (NaCl, ≥99.9%), hydrochloric acid (HCl, 12.0 M) polydimethylsiloxane (PDMS), polytetrafluoroethylene (PTFE) tube, and all the other reagents used to synthesize the photoacid were purchased from Sigma-Aldrich (St. Louis, MO, USA). SiN membrane was from Norcada, Inc. (Edmonton, Canada).

### Synthesis of the Photoacid

A photoacid with protonated merocyanine (MEH) structure was synthesized. Scheme of the synthesis process was shown in Supplementary Figure 1. pr-MEH was synthesized following a literature method (Mason et al., 2005). 2,3,3-trimethylindolenine (1.59 g, 10 mmol) was added to propane sultone (1.22 g, 10 mmol). The mixture was stirred at 90°C overnight under nitrogen atmosphere. The crude product was filtered, washed with cold diethyl ether, and dried in vacuo to get pr-MEH (2.50 g, 85%). For the synthesis of MEH, pr-MEH (1 g, 3.6 mmol) and 2-hydroxybenzaldehyde (0.48 g, 3.9 mmol) were added into anhydrous ethanol (15 mL). The mixture was allowed to reflux overnight. The orange solid of MEH was obtained by filtration (1 g, 78%). ^1^H NMR (400 MHz, DMSO-d_6_, δ, ppm): δ = 11.05 (s, 1H), 8.59 (d, 1 H), 8.25 (d, 1 H), 8.01 (d, 1 H), 7.86 (m, 2 H), 7.61 (m, 2 H), 7.44 (t, 1H), 7. 03 (d, 1 H), 6.95 (t, 1 H), 4.79 (t, 2 H), 2.64 (t, 2 H), 2.13 (m, 2 H), 1.78 (s, 6 H) (see Supplementary Figure 2). ToF-SIMS analysis of the product was shown in Supplementary Figure 3. The maximum absorption wavelength in UV-vis spectra was ~ 425 nm (Supplementary Figure 4).

### Fabrication of the Microfluidic Reactor

Fabrication of the microfluidic reactor was described in our previous paper (Liu et al., 2019). Briefly, a micro-chamber was made by pouring PDMS on a silicon mode fabricated by soft lithography. The as-prepared PDMS block was sealed with a silicon-framed silicon nitride window *via* air plasma. An inlet and outlet were drilled through the PDMS block. After injection of sample solution, the two ends were sealed by a PEEK union. After that, the microfluidic reactor was introduced into the high vacuum chamber for subsequent *in-situ* liquid ToF-SIMS measurements.

### Hybrid Light/ToF-SIMS Instrumentation

A ToF-SIMS V spectrometer (IONTOF GmbH, Germany) equipped with a 30 keV ${\text{Bi}}_{3}^{+}$ primary ion beam was used for *in-situ* liquid ToF-SIMS analysis. For *in-situ* liquid ToF-SIMS analysis, the target current, and lateral resolution were adjusted to 0.35 pA and 200 nm, respectively. As shown in Supplementary Figure 5A, a 100 nm thick SiN membrane supported on a silicon frame (window size 0.5 × 0.5 mm^2^) was irreversibly bonded with the PDMS block containing a 200 × 300 μm (width × depth) channel to form the detection area. For sampling, a liquid-vacuum interface was formed by drilling a ~2 μm pore through the SiN membrane by primary ion beam before continuous signal recording of the liquid-vacuum interface. The dynamic depth profiling by ToF-SIMS for 0.5 mM MEH solution in the positive mode was used to monitoring the submicropore condition at the SiN membrane surface. As presented in Supplementary Figure 5B, SiN membrane was punched through at around 295 s with the dramatically increased intensity of [MEH+H]^+^ and decreased signal of Si_2_N^+^. The ultrahigh surface tension of liquid water in a sub micrometer pore can confine the liquid water in the microfluidic chip. Thus, a liquid-vacuum interface used for the *in-situ* liquid detection is formed under high vacuum condition. When the liquid-vacuum interface at the confined pore was sable, the pulse width was adjusted from 160 to 80 ns immediately for better mass resolution. Then the mass spectrum signal of the water clusters from 0.5 mM MEH solution surface was acquired in the microfluidic reactor (see Supplementary Figure 5C). During analysis, a flashlight (Skyfire, white light with an optical filter at 420 nm) with a power of 12 W was used to irradiate the microfluidic chip. By adjusting the flashlight, real-time ToF-SIMS analysis of light-activated reaction was realized. The obtained ToF-SIMS spectra were calibrated using C^+^, ${\text{CH}}_{2}^{+}$, C_2_${\text{H}}_{5}^{+}$, C_3_${\text{H}}_{3}^{+}$ for positive mass spectra and C^−^, ${\text{CH}}_{2}^{-}$, ${\text{C}}_{2}^{-}$, ${\text{C}}_{3}^{-}$ for negative mass spectra, respectively. The two-dimensional (2D) images of Si^+^, [MEH+H]^+^ and (H_2_O)_3_H^+^ can be used to estimate the pore size. Besides, in order to improve the signal to noise (S/N) of mass spectrum, the mass spectra data could be reconstructed at the selected ROI area of the pore center in 2D images (Supplementary Figure 5D).

### DFT Simulation

The calculations in this work were performed by the Gaussian 09 suite of programs (Frisch, 2009). The geometries of the protonated water clusters under study were fully optimized by the hybrid M06-2x functional, in which developed by Zhao and Truhlar, has proved to be reliable in the description of various types of non-covalent interactions (Zhao and Truhlar, 2008). The Dunning's basis set aug-cc-pVTZ, was utilized for all the atoms (Kendall et al., 1992). No symmetry or geometry constraint was applied during the optimizations. All the optimized-geometries were corroborated to be factual minima on the potential energy surface by means of frequency calculation at the same theoretical level.

The interaction energy (Δ*E*) between hydronium ion and waters for per water molecule was evaluated as

$$\Delta E_{per}=\frac{1}{n}(E_{systerm}-E_{H_{3}O^{+}}-E_{nH_{2}O})$$

Where *E*_system_ is the energy of the optimized whole system; *E*_H3O+_ and *E*_nH2O_ are the total electronic energies of the hydronium ion and waters, respectively, which kept in the same structure as that in the optimized whole system. The *n* denotes the number of waters.

## Results and Discussion

### ToF-SIMS Real-Time Analysis of the Light-Activated Proton Dissociation

**Figure 1:**
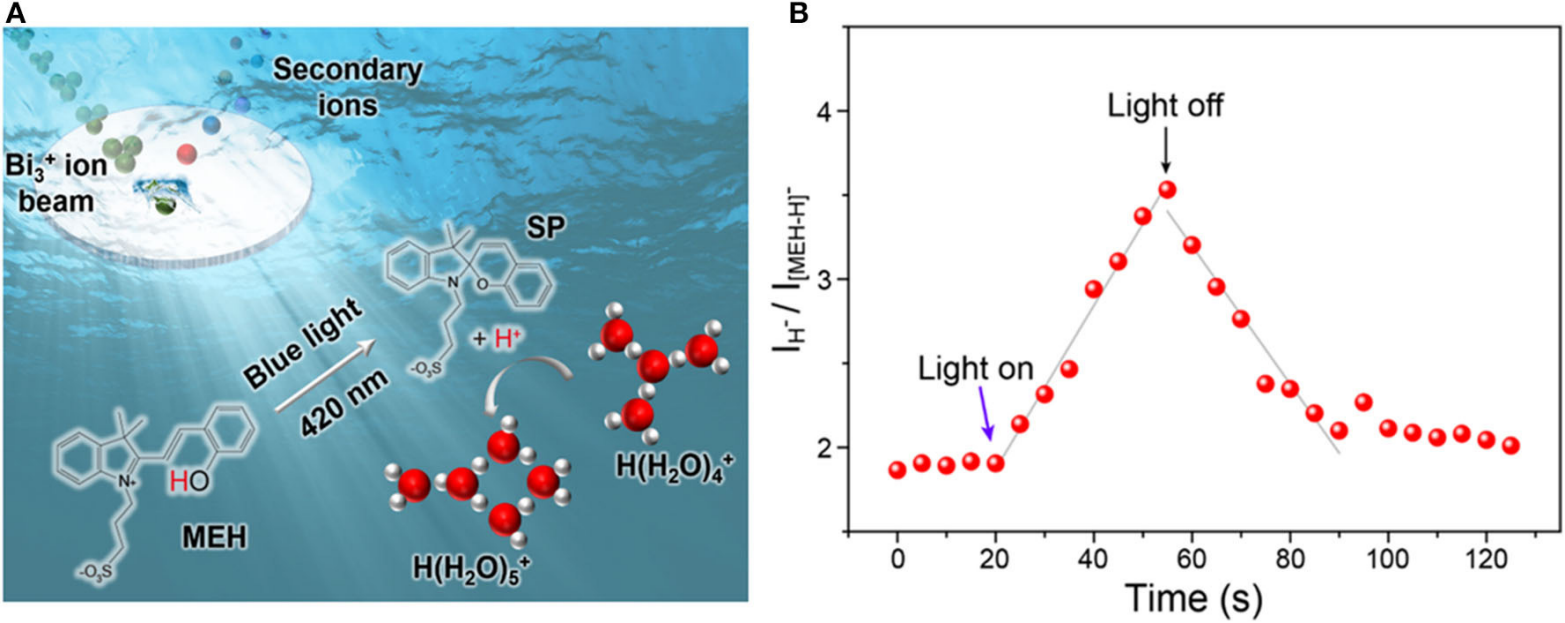
**(A)** Schematic illustration of the *in-situ* liquid ToF-SIMS analysis of water clusters adjusted by MEH. **(B)** Time-dependent intensity ratio of proton (m/z 1, H^−^) and MEH (m/z 384, [MEH-H]^−^) mediated by blue light (420 nm).

A photoacid with protonated merocyanine (MEH) structure was used as a model to *in-situ* generate free protons in aqueous solutions. Based on previous studies (Shi et al., 2011; Liao, 2017), MEH was the first reported metastable-state photoacid. It was known that upon illumination of blue light, one proton of MEH could be released and the MEH will turn into SP. When the light was turned off, the less stable SP will convert back into MEH (Figure 1A). The mechanism of light-activated proton release is described in Supplementary Figure 6. Upon irradiation with visible light (420 nm), MEH firstly convert into the *cis*-MEH and then *cis*-ME after releasing a proton. Once the proton was transferred, *cis*-ME wound turn into SP via a nucleophilic ring closing reaction. SP tends to return to its original state (MEH) immediately in the dark, which means the whole process is reversible. To directly monitor the light-activated proton dissociation, *in-situ* liquid ToF-SIMS in combination with a 420 nm light source was used. A ~2 μm pore on the SiN membrane of the microfluidic cell was drilled *in-situ* by the primary ion beam of ToF-SIMS. Thus, a pore-confined liquid-vacuum interface was formed for light illumination as well as direct ToF-SIMS measurement (Figure 1A). As shown in Figure 1B, the intensity ratio between proton (m/z 1) and MEH (m/z 384, [M-H]^−^) started to increase linearly upon light illumination. When the light was turned off at ~55 s, the ratio started to decrease. It takes about 35 s for the ratio to reach the baseline, which is quite similar to the light illumination process (from 20 to 55 s). The results indicated that the kinetic constant of proton dissociation and recombination process were similar. In addition, the result demonstrated the capability of *in-situ* liquid ToF-SIMS for real-time monitoring of proton transfer process mediated by light.

### Dynamic Change of Water Clusters Mediated by Photoacid

**Figure 2:**
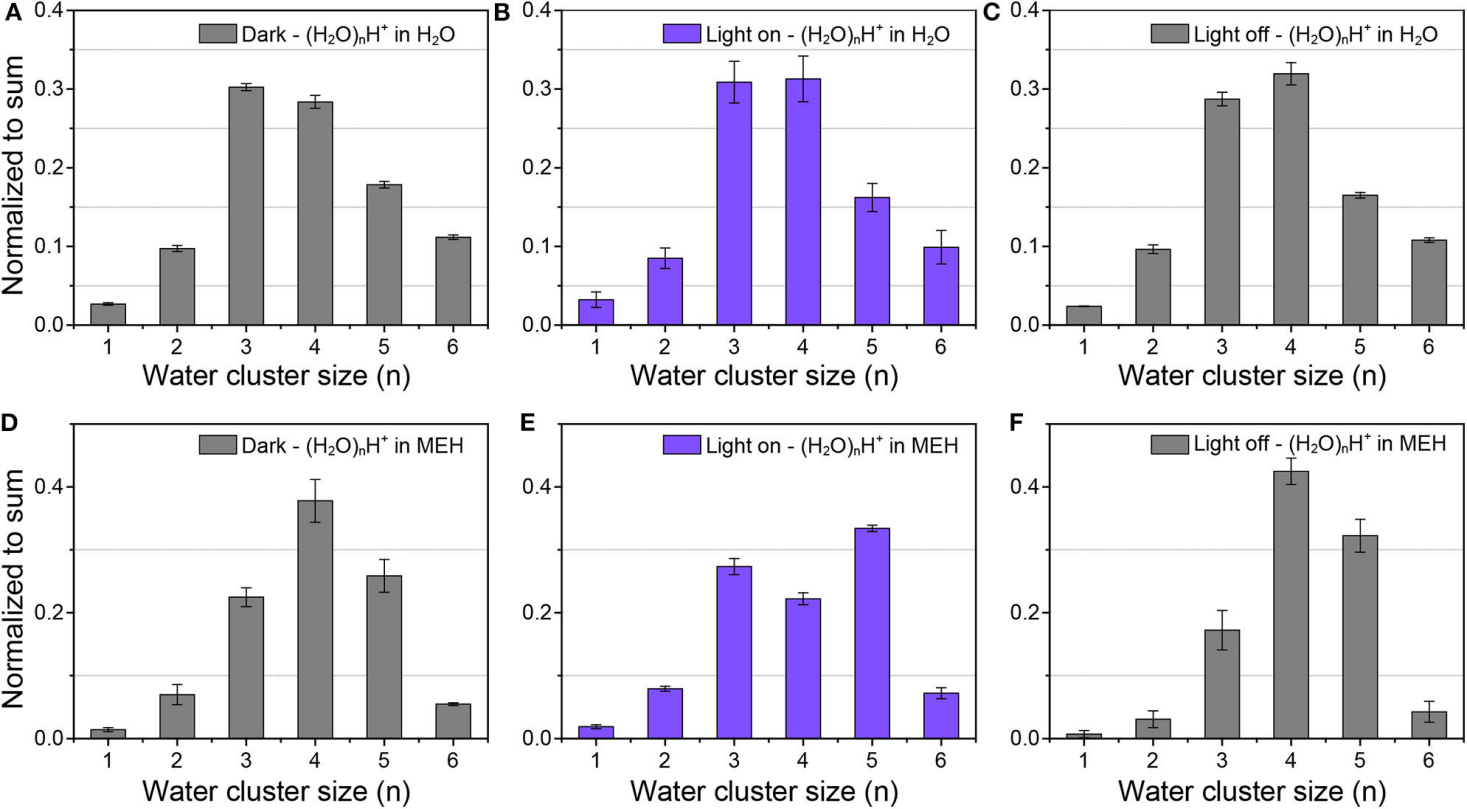
Protonated water cluster size distributions in **(A–C)** pure water and **(D–F)** 0.5 mM MEH solutions in dark environment **(A,D)**, under light illumination **(B,E)**, and after light was turned off **(C,F)**, respectively.

The dissociation of protons in aqueous acid solutions changed the micro-environment of the water structure, which could lead to the change of hydrogen bonding networks as well as the size distribution of protonated water clusters. The effect of proton released from organic photoacid on HBs and water structures was real-time measured by *in-situ* liquid ToF-SIMS. Before analysis of the light-mediated water cluster distribution of MEH, we firstly measured the water cluster size distribution of pure water as a control. As shown in Figures 2A–C, the predominant water clusters in pure water were (H_2_O)_3_H^+^ and (H_2_O)_4_H^+^. In pure water, the peak intensity order was: (H_2_O)_3_H^+^ > (H_2_O)_4_H^+^ > (H_2_O)_5_H^+^ > (H_2_O)_2_H^+^ ≈ (H_2_O)_6_H^+^. The result was in good agreement with a previous computational study by Natarajan et al. (2015). In their study, both neural network (NN) potential and density-functional theory (DFT) calculations revealed that the order of binding energies E_bind_ per water monomer of the protonated water clusters was: (H_2_O)_3_H^+^ < (H_2_O)_4_H^+^ < (H_2_O)_5_H^+^ < (H_2_O)_2_H^+^ < (H_2_O)_6_H ^+^, indicating an energetic stability order of (H_2_O)_3_H^+^ > (H_2_O)_4_H^+^ > (H_2_O)_5_H^+^ > (H_2_O)_2_H^+^ > (H_2_O)_6_H^+^. For better understanding of the structure of protonated water cluster, the potential geometries of (H_2_O)_3_H^+^, (H_2_O)_4_H^+^ and (H_2_O)_5_H^+^ water clusters were optimized and presented in Supplementary Figure 7. Besides, the order of interaction energy (ΔE, kcal/mol) between hydronium ion and waters for per water molecule was: (H_2_O)_3_H^+^ < (H_2_O)_4_H^+^ < (H_2_O)_5_H^+^, which was consistent with the previous simulation results and our experiment result. Additionally, no obvious differences were found in dark or light illumination conditions. The results demonstrated negligible influence of light illumination on the water cluster size distribution of pure water, including (H_2_O)_n_H^+^ and (H_2_O)_n_OH^−^ water clusters (see Supplementary Figures 8A–C). However, after addition of 0.5 mM MEH into pure water, the proportion of (H_2_O)_3_H^+^ significantly decreased, while that for (H_2_O)_4_H^+^ and (H_2_O)_5_H^+^ increased (Figure 2D). According to previous report, MEH was a weak acid with a pK_a_ of ~7.8. The pH of 0.5 mM MEH was ~5.5 (under dark environment) (Gutberlet et al., 2009). The increase of free proton concentration could lead to a strengthened hydrogen bonding networks and thus increase the ratio of relatively larger water clusters. Another important reason for this change is the disturbing of organic molecules on the hydrogen bonding networks of water. It was reported that small purely hydrophobic solutes tend to strengthen nearby water hydrogen bonding networks (Grdadolnik et al., 2017). As shown in Figure 1A, the structure of MEH was relatively complicated with various hydrophobic functional groups, which could strengthen the local hydrogen bonding networks. Therefore, the ratio between larger water clusters and smaller clusters tend to increase.

As displayed in Figures 2D–F, in dark environment, the dominant protonated water cluster in MEH solution was (H_2_O)_4_H^+^. Upon light illumination, the ratio between (H_2_O)_4_H^+^ and (H_2_O)_5_H^+^ obviously decreased. (H_2_O)_5_H^+^ became the most dominant water cluster. After the light was turned off, the dominant specie changed back to (H_2_O)_4_H^+^. These results indicated the fact that the increased free protons in aqueous solution could lead to the formation of lager protonated water clusters. Moreover, as shown in Supplementary Figures 8D–F, the dominant hydroxide water cluster in MEH solution was (H_2_O)_2_OH^−^ and (H_2_O)_3_OH^−^ in dark. While under light illumination, the dominant specie switched into (H_2_O)_4_OH^−^. And as expected, when the light was turned off, the dominant species changed back into (H_2_O)_2_OH^−^ and (H_2_O)_3_OH^−^. These results further demonstrated that the hydrogen bonding networks were further strengthened upon increasing the concentration of free protons. This result was further evidenced by pH-dependent changes of protonated water clusters in inorganic acid solutions.

**Figure 3:**
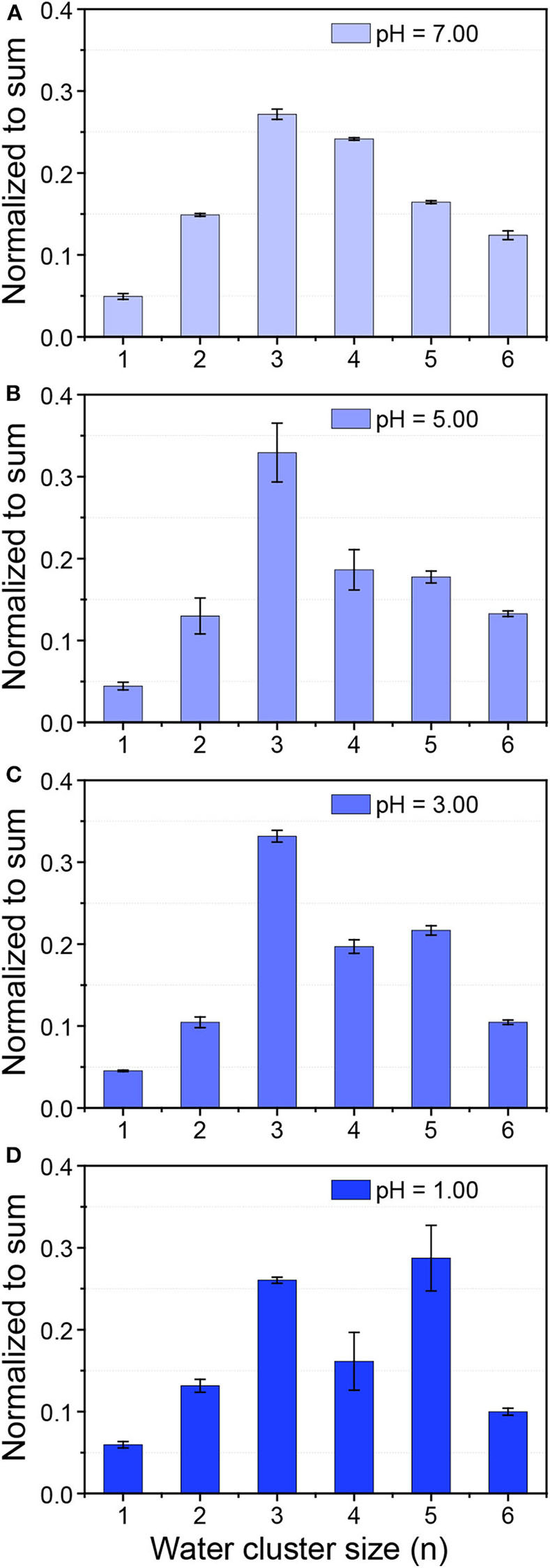
Size distribution of protonated water clusters in 0.01 mM NaCl solution with a pH of 7.00 **(A)** and HCl solution with a pH of 5.00 **(B)**, 3.00 **(C)**, and 1.00 **(D)**.

Here hydrochloride acid (HCl) was chosen since it was known that Cl^−^ has negligible effects on HB networks and water clusters (Näslund et al., 2005; Liu et al., 2019). *In-situ* liquid ToF-SIMS measurement of the size distributions of protonated water clusters in HCl solutions under different pH were shown in Figure 3. It was clear that with the decrease of pH, the intensity ratio of (H_2_O)_4_H^+^ gradually decreased while that for (H_2_O)_5_H^+^ increased. When pH was decreased to one, the signal intensity of (H_2_O)_5_H^+^ even exceeded (H_2_O)_3_H^+^, which was the dominant protonated water cluster under neutral pH. That demonstrated the formation of relatively larger protonated water clusters in concentrated acid solution. This is in good agreement with previous observations that an increase of the acid concentration lead to the increase of the average number of donating HBs per water molecule by X-ray Raman scattering (XRS) and XAS (Cavalleri et al., 2006; Chen et al., 2013). However, in those studies, it was not until the concentration of hydrochloric acid increased to several M that the spectra started to change. Our results provided direct molecular evidence that the structure of water was changed even when the concentration of hydrochloric acid was lower than 0.1 M.

**Figure 4:**
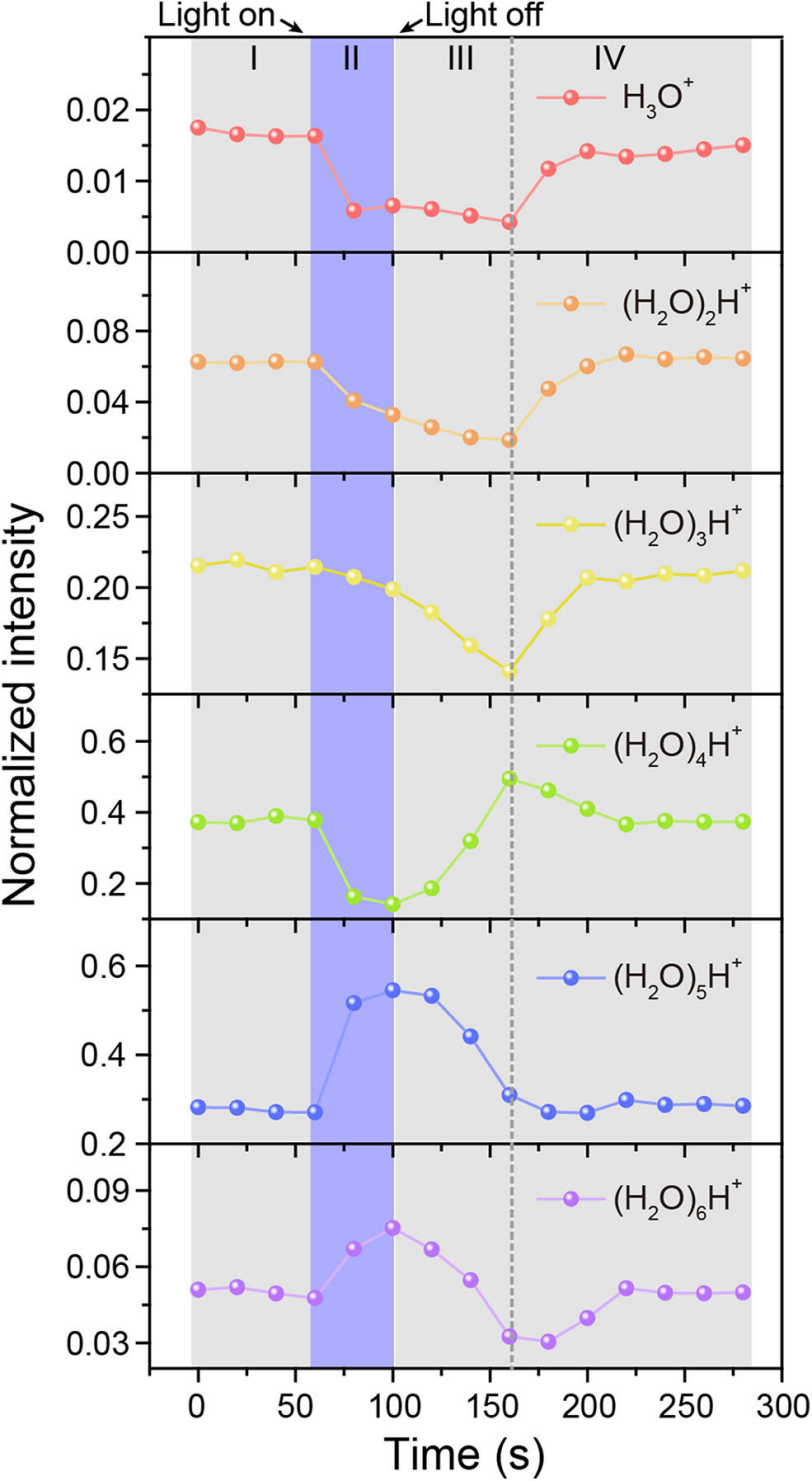
Time-resolved intensity changes of protonated water clusters. MEH solution was kept in dark (I), under illumination (II), and in dark again after illumination (III and IV), respectively.

For real-time monitoring the dynamic process of protonated water cluster size changes with time, the mass spectrum data was reconstructed every 20 s to obtain the mass spectrum peak area of water cluster signals. The normalized intensity changes of each water cluster ((H_2_O)_n_H^+^, *n* = 1–6) with time was presented in Figure 4. The dominant protonated water cluster in MEH solution under dark environment was (H_2_O)_4_H^+^ (Region I in Figure 4). Once the light was turned on (Region II), the ratio of smaller water clusters of (H_2_O)_n_H^+^ (*n* = 1–4) decreased immediately with the increased concentration of proton, while the larger ones of (H_2_O)_5_H^+^ and (H_2_O)_6_H^+^ increased, especially for (H_2_O)_5_H^+^, whose ratio is about 1.7 times that in dark environment. Interestingly, when the light was turned off, the concentration of proton decreased, the large water clusters of (H_2_O)_5_H^+^ and (H_2_O)_6_H^+^ firstly changed into (H_2_O)_4_H^+^ (see region III). After that, (H_2_O)_4_H^+^ changed into smaller water clusters of (H_2_O)_n_H^+^(*n* = 1–3) and then the size distribution of water clusters gradually return to the initial level (Region IV). For better understanding of the above process, the schematic diagram of water cluster structure changes mediated by photoacid under dark and illumination conditions were shown in Supplementary Figure 9.

### Proton Accumulation at the Pore-Confined Liquid-Vacuum Interface

**Figure 5:**
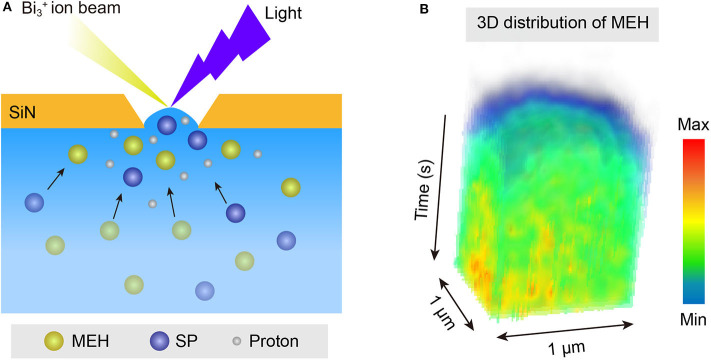
**(A)** Schematic illustration of the accumulation of MEH and dissociated protons at the pore-confined liquid-vacuum interface. **(B)** ToF-SIMS continuous mapping of MEH at the pore-confined liquid-vacuum interface, with a region of interest (ROI) for 1 × 1 μm.

An interesting phenomenon is that the size distribution of protonated water clusters in MEH under light illumination (Figure 2E) was quite similar to that in HCl solution with pH = 1 (Figure 3D). Previous report demonstrated that even under complete proton dissociation, the pH of 0.5 mM MEH solution was 3.2 (Shi et al., 2011). However, the results in light illuminated MEH was closer to the results of HCl with pH = 1. According to the abnormal results, we speculate that in the light-mediated proton dissociation, proton tends to accumulate at the pore-confined liquid-vacuum interface. Thus, the local concentration of protons at the sampling area significantly increased, leading to a further strengthened hydrogen bonding networks as well as larger water clusters at the liquid-vacuum interface. It was well-known that the hydrophobic parts of a complicated molecule tend to approach the gas phase at a liquid-gas interface (Björneholm et al., 2016). Thus, in this case, it is reasonable to hypothesis the accumulation of MEH at the liquid-vacuum interface (Figure 5A). ToF-SIMS 3D mapping of MEH at the center of liquid-vacuum interface at the very beginning of pore formation was conducted to directly visualize the concentration change of MEH at the interface as a function of time (Figure 5B). It was clearly observed that the concentration of MEH continuously increased along with time, which verified the accumulation of MEH at the liquid-vacuum interface. As a result, the concentration of dissociated protons significantly increased at the interface, indicating a lower pH at the interface than in the bulk.

## Conclusion

In this work, the effects of inorganic and organic acids on water structure and hydrogen bonding networks in aqueous solutions were investigated by *in-situ* liquid ToF-SIMS. The results revealed that higher concentration of dissociated protons lead to an enhanced local hydrogen bonding networks as well as relatively larger protonated water clusters. In combination with a light illumination system, *in-situ* liquid ToF-SIMS holds the ability to real-time analyze light-activated proton dissociation and the resulting changes of protonated water clusters. In addition, the accumulation of organic photoacid and dissociated protons at the pore-confined liquid-vacuum interface caused by the affinity of organic molecules to the liquid-vacuum interface was observed. These results provided direct molecular evidence of the interaction between protons and water, which was of significance for the fundamental investigations of related atmospheric and physiochemical processes. Besides, the light-illumination system combined with *in-situ* liquid ToF-SIMS method shows great potential in the study of light-sensitized processes in aqueous solutions.

## Data Availability Statement

The raw data supporting the conclusions of this article will be made available by the authors, without undue reservation.

## Author Contributions

XH and Y-TL designed this work. Y-YL conducted the experiments. XH and Y-YL wrote this paper. ZZ and JZ synthesized the photoacid. SZ performed the DFT simulation. PH gave constructive suggestions in addressing the reviewers' comments. All authors contributed to the article and approved the submitted version.

## Conflict of Interest

The authors declare that the research was conducted in the absence of any commercial or financial relationships that could be construed as a potential conflict of interest.

## References

[B1] Agmon N. (1995). The grotthuss mechanism. Chem. Phys. Lett. 244, 456–462. 10.1016/0009-2614(95)00905-J

[B2] Badalyan A. Stahl S. S. (2016). Cooperative electrocatalytic alcohol oxidation with electron-proton-transfer mediators. Nature 535, 406–410. 10.1038/nature18008 27350245

[B3] Björneholm O. Hansen M. H. Hodgson A. Liu L.-M. Limmer D. T. Michaelides A.. (2016). Water at interfaces. Chem. Rev. 116, 7698–7726. 10.1021/acs.chemrev.6b00045 27232062

[B4] Bouazizi S. Nasr S. Jaîdane N. Bellissent-Funel M.-C. (2006). Local order in aqueous NaCl solutions and pure water: X-ray scattering and molecular dynamics simulations study. J. Phys. Chem. B 110, 23515–23523. 10.1021/jp0641583 17107207

[B5] Burnham C. J. Petersen M. K. Day T. J. Iyengar S. S. Voth G. A. (2006). The properties of ion-water clusters. II. Solvation structures of Na+, Cl-, and H+ clusters as a function of temperature. J. Chem. Phys. 124:024327. 10.1063/1.2149375 16422603

[B6] Cavalleri M. Näslund L.-Å. Edwards D. C. Wernet P. Ogasawara H. Myneni S.. (2006). The local structure of protonated water from x-ray absorption and density functional theory. J. Chem. Phys. 124:194508. 10.1063/1.2199828 16729826

[B7] Chen C. Huang C. Waluyo I. Nordlund D. Weng T.-C. Sokaras D.. (2013). Solvation structures of protons and hydroxide ions in water. J. Chem. Phys. 138:154506. 10.1063/1.4801512 23614429

[B8] Chen M. Zheng L. Santra B. Ko H.-Y. DiStasio R. A. Jr Klein M. L.. (2018). Hydroxide diffuses slower than hydronium in water because its solvated structure inhibits correlated proton transfer. Nat. Chem. 10, 413–419. 10.1038/s41557-018-0010-2 29531374

[B9] Chen Y. Okur H. I. Gomopoulos N. Macias-Romero C. Cremer P. S. Petersen P. B.. (2016). Electrolytes induce long-range orientational order and free energy changes in the H-bond network of bulk water. Sci. Adv. 2:e1501891. 10.1126/sciadv.1501891 27152357 PMC4846452

[B10] Daldrop J. O. Saita M. Heyden M. Lorenz-Fonfria V. A. Heberle J. Netz R. R. (2018). Orientation of non-spherical protonated water clusters revealed by infrared absorption dichroism. Nat. Comm. 9, 1–7. 10.1038/s41467-017-02669-9 29358659 PMC5778031

[B11] Di Luca A. Gamiz-Hernandez A. P. Kaila V. R. (2017). Symmetry-related proton transfer pathways in respiratory complex I. Proc. Natl. Acad. Sci. U. S. A. 114, E6314–E6321. 10.1073/pnas.1706278114 28716925 PMC5547640

[B12] Ding C.-G. Laasonen K. Laaksonen A. (2003). Two sulfuric acids in small water clusters. J. Phys. Chem. A 107, 8648–8658. 10.1021/jp022575j

[B13] Ellmer M. A. Sener C. Gallo J. M. Luterbacher J. S. Alonso D. M. Dumesic J. A. (2014). Solvent effects in acid-catalyzed biomass conversion reactions. Angew. Chemie. Int. Ed. 53, 11872–11875. 10.1002/anie.201408359 25214063

[B14] Frisch M. J. (2009). Gaussian 09. Available online at: http://www.gaussian.com/ (accessed May 05, 2020).

[B15] Fu C. Xu J. Boyer C. (2016). Photoacid-mediated ring opening polymerization driven by visible light. Chem. Comm. 52, 7126–7129. 10.1039/C6CC03084J 27167862

[B16] Garczarek F. Gerwert K. (2006). Functional waters in intraprotein proton transfer monitored by FTIR difference spectroscopy. Nature 439, 109–112. 10.1038/nature04231 16280982

[B17] Gould N. S. Li S. Cho H. J. Landfield H. Caratzoulas S. Vlachos D. G.. (2020). Understanding solvent effects on adsorption and protonation in porous catalysts. Nat. Commun. 11, 1–13. 10.1038/s41467-020-14860-6 32103007 PMC7044222

[B18] Grdadolnik J. Merzel F. Avbelj F. (2017). Origin of hydrophobicity and enhanced water hydrogen bond strength near purely hydrophobic solutes. Proc. Natl. Acad. Sci. U. S. A. 114, 322–327. 10.1073/pnas.1612480114 28028244 PMC5240716

[B19] Guo J.-X. Zhou T. Xu B. Zhu S.-F. Zhou Q.-L. (2016). Enantioselective synthesis of α-alkenyl α-amino acids via N–H insertion reactions. Chem. Sci. 7, 1104–1108. 10.1039/C5SC03558A 29910866 PMC5975786

[B20] Gutberlet A. Schwaab G. Birer Ö. Masia M. Kaczmarek A. Forbert H.. (2009). Aggregation-induced dissociation of HCl (H_2_O)_4_ below 1 K: the smallest droplet of acid. Science 324, 1545–1548. 10.1126/science.1171753 19541993

[B21] Headrick J. M. Diken E. G. Walters R. S. Hammer N. I. Christie R. A. Cui J.. (2005). Spectral signatures of hydrated proton vibrations in water clusters. Science 308, 1765–1769. 10.1126/science.1113094 15961665

[B22] Johns V. K. Patel P. K. Hassett S. Calvo-Marzal P. Qin Y. Chumbimuni-Torres K. Y. (2014). Visible light activated ion sensing using a photoacid polymer for calcium detection. Anal. Chem. 86, 6184–6187. 10.1021/ac500956j 24893213

[B23] Kendall R. A. Dunning T. H. Jr Harrison R. J. (1992). Electron affinities of the first-row atoms revisited. Systematic basis sets and wave functions. J. Chem. Phys. 96, 6796–6806. 10.1063/1.462569

[B24] Lee C. Sosa C. Planas M. Novoa J. J. (1996). A theoretical study of the ionic dissociation of HF, HCl, and H_2_S in water clusters. J. Chem. Phys. 104, 7081–7085. 10.1063/1.471426

[B25] Li H. Kong X. Jiang L. Liu Z. (2019). The size-dependent formation of an ion pair in HSO_4_-(H_2_O)_n_: a molecular model for probing the micro-solvation of acid dissociation. J. Phys. Chem. Lett. 10, 2162–2169. 10.1021/acs.jpclett.9b00699 30995405

[B26] Li Z. Lazaridis T. (2006). Thermodynamics of buried water clusters at a protein– ligand binding interface. J. Phys. Chem. B 110, 1464–1475. 10.1021/jp056020a 16471698

[B27] Liao Y. (2017). Design and applications of metastable-state photoacids. Acc. Chem. Res. 50, 1956–1964. 10.1021/acs.accounts.7b00190 28692282

[B28] Liu Y.-Y. Ying Y.-L. Hua X. Long Y.-T. (2018). *In-situ* discrimination of the water cluster size distribution in aqueous solution by ToF-SIMS. Sci. China Chem. 61, 159–163. 10.1007/s11426-017-9180-1

[B29] Liu Y.-Y. Zhang S.-Z. Ying Y.-L. Xia H.-L. Hua X. Long Y.-T. (2019). Ion-specific effects on hydrogen bond network at a submicropore confined liquid-vacuum interface: an *in situ* liquid ToF-SIMS study. J. Phys. Chem. Lett. 10, 4935–4941. 10.1021/acs.jpclett.9b02047 31403310

[B30] Marcus Y. Hefter G. (2006). Ion pairing. Chem. Rev. 106, 4585–4621. 10.1021/cr040087x 17091929

[B31] Mason S. J. Hake J. L. Nairne J. Cummins W. J. Balasubramanian S. (2005). Solid-phase methods for the synthesis of cyanine dyes. J. Orga. Chem. 70, 2939–2949. 10.1021/jo0479415 15822952

[B32] Meiboom S. (1961). Nuclear magnetic resonance study of the proton transfer in water. J. Chem. Phys. 34, 375–388. 10.1063/1.1700960

[B33] Miyazaki M. Fujii A. Ebata T. Mikami N. (2004). Infrared spectroscopic evidence for protonated water clusters forming nanoscale cages. Science 304, 1134–1137. 10.1126/science.1096037 15118121

[B34] Mohammed O. F. Pines D. Dreyer J. Pines E. Nibbering E. T. (2005). Sequential proton transfer through water bridges in acid-base reactions. Science 310, 83–86. 10.1126/science.1117756 16210532

[B35] Mohammed O. F. Pines D. Nibbering E. T. Pines E. (2007). Base-induced solvent switches in acid–base reactions. Angew. Chemie. Int. Ed. 46, 1458–1461. 10.1002/anie.200603383 17212371

[B36] Näslund L.-Å. Edwards D. C. Wernet P. Bergmann U. Ogasawara H. Pettersson L. G.. (2005). X-ray absorption spectroscopy study of the hydrogen bond network in the bulk water of aqueous solutions. J. Phys. Chem. A 109, 5995–6002. 10.1021/jp050413s 16833935

[B37] Natarajan S. K. Morawietz T. Behler J. (2015). Representing the potential-energy surface of protonated water clusters by high-dimensional neural network potentials. Phys. Chem. Chem. Phys. 17, 8356–8371. 10.1039/C4CP04751F 25436835

[B38] Park S. Fayer M. D. (2007). Hydrogen bond dynamics in aqueous NaBr solutions. Proc. Natl. Acad. Sci. U. S. A. 104, 16731–16738. 10.1073/pnas.0707824104 17940023 PMC2040434

[B39] Park S. Yoo B. Pyo J. Kim M. S. Jang D. (2012). Anomalous acid-base equilibria in biologically relevant water nanopools. Bull. Korean Chem. Soc. 33, 3493–3496. 10.5012/bkcs.2012.33.10.3493

[B40] Shalit A. Ahmed S. Savolainen J. Hamm P. (2017). Terahertz echoes reveal the inhomogeneity of aqueous salt solutions. Nat. Chem. 9, 273–278. 10.1038/nchem.2642 28221356

[B41] Shi R. Huang X. Su Y. Lu H.-G. Li S.-D. Tang L.. (2017). Which density functional should be used to describe protonated water clusters? J. Phys. Chem. A 121, 3117–3127. 10.1021/acs.jpca.7b00058 28383918

[B42] Shi Z. Peng P. Strohecker D. Liao Y. (2011). Long-lived photoacid based upon a photochromic reaction. J. Am. Chem. Soc. 133, 14699–14703. 10.1021/ja203851c 21823603

[B43] Stirnemann G. Wernersson E. Jungwirth P. Laage D. (2013). Mechanisms of acceleration and retardation of water dynamics by ions. J. Am. Chem. Soc. 135, 11824–11831. 10.1021/ja405201s 23865559

[B44] Waluyo I. Nordlund D. Bergmann U. Schlesinger D. Pettersson L. G. Nilsson A. (2014). A different view of structure-making and structure-breaking in alkali halide aqueous solutions through x-ray absorption spectroscopy. J. Chem. Phys. 140:244506. 10.1063/1.4881600 24985653

[B45] Wang L. Zhao J. Fang H. (2008). Water clusters confined in nonpolar cavities by ab initio calculations. J. Phys. Chem. C 112, 11779–11785. 10.1021/jp8048185

[B46] Yang L. Yu X.-Y. Zhu Z. Iedema M. J. Cowin J. P. (2011). Probing liquid surfaces under vacuum using SEM and ToF-SIMS. Lab. Chip 11, 2481–2484. 10.1039/c0lc00676a 21670825

[B47] Ye S. Nihonyanagi S. Uosaki K. (2001). Sum frequency generation (SFG) study of the pH-dependent water structure on a fused quartz surface modified by an octadecyltrichlorosilane (OTS) monolayer. Phys. Chem. Chem. Phys. 3, 3463–3469. 10.1039/b101673n

[B48] Yucknovsky A. Mondal S. Burnstine-Townley A. Foqara M. Amdursky N. (2019). Use of photoacids and photobases to control dynamic self-assembly of gold nanoparticles in aqueous and nonaqueous solutions. Nano Lett. 19, 3804–3810. 10.1021/acs.nanolett.9b00952 31124686

[B49] Zhao L. Ma K. Yang Z. (2015). Changes of water hydrogen bond network with different externalities. Int. J. Mol. Sci. 16, 8454–8489. 10.3390/ijms16048454 25884333 PMC4425091

[B50] Zhao Y. Truhlar D. G. (2008). The M06 suite of density functionals for main group thermochemistry, thermochemical kinetics, noncovalent interactions, excited states, and transition elements: two new functionals and systematic testing of four M06-Class functionals and 12 other functionals. Theory. Chem. Acc. 120, 215–241. 10.1007/s00214-007-0310-x

