# Supplementary material for: pH-Dependent Water Clusters in Photoacid Solution: Real-Time Observation by ToF-SIMS at a Submicropore Confined Liquid-Vacuum Interface
Source: Front Chem. 2020 Aug 21;8:731. doi: 10.3389/fchem.2020.00731 (PMC7472850; doi:10.3389/fchem.2020.00731)
Supplement: Supplementary file 1 [file Table_1.DOCX]

Supplementary Material


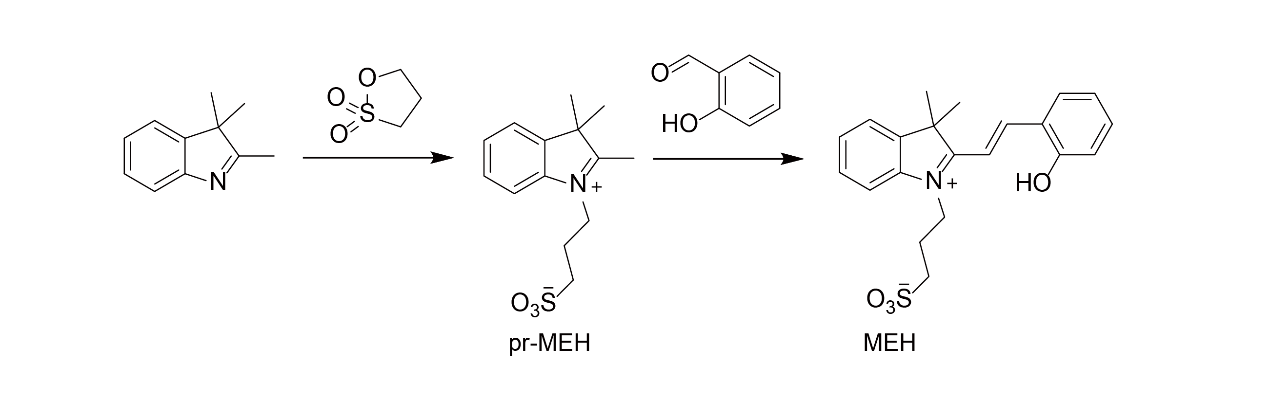


**Supplementary Figure 1.** Scheme of the synthesis process of MEH.


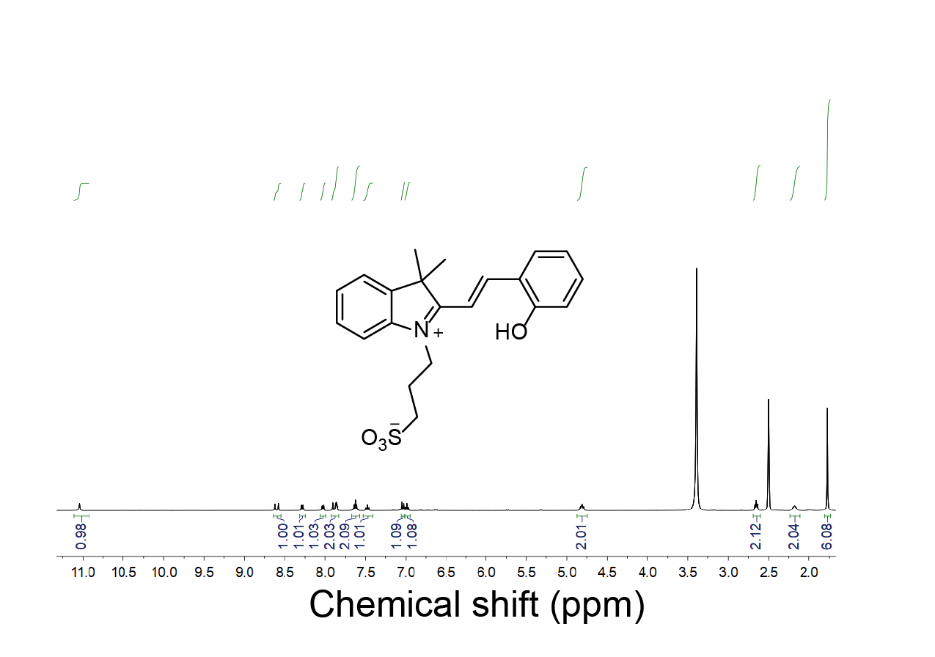


**Supplementary Figure 2.** ^1^H NMR spectrum of the synthesized MEH.


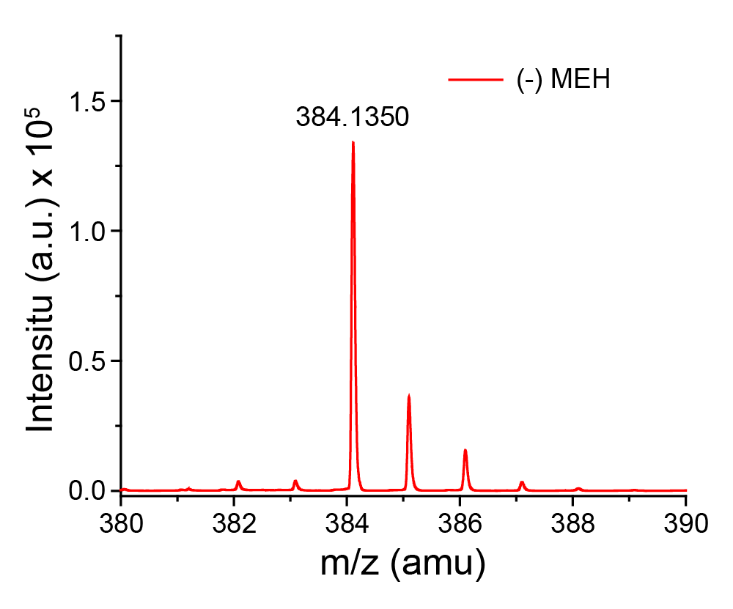


**Supplementary Figure 3.** ToF-SIMS analysis of the synthesized MEH.


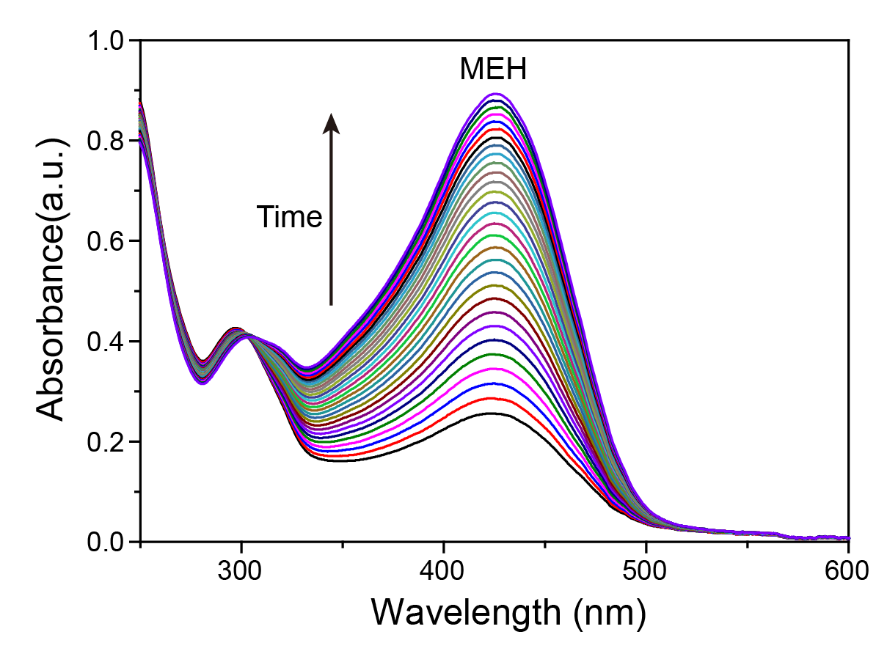


**Supplementary Figure 4.** UV-vis spectra of the solution of synthesized MEH 20-170 s after irradiation.


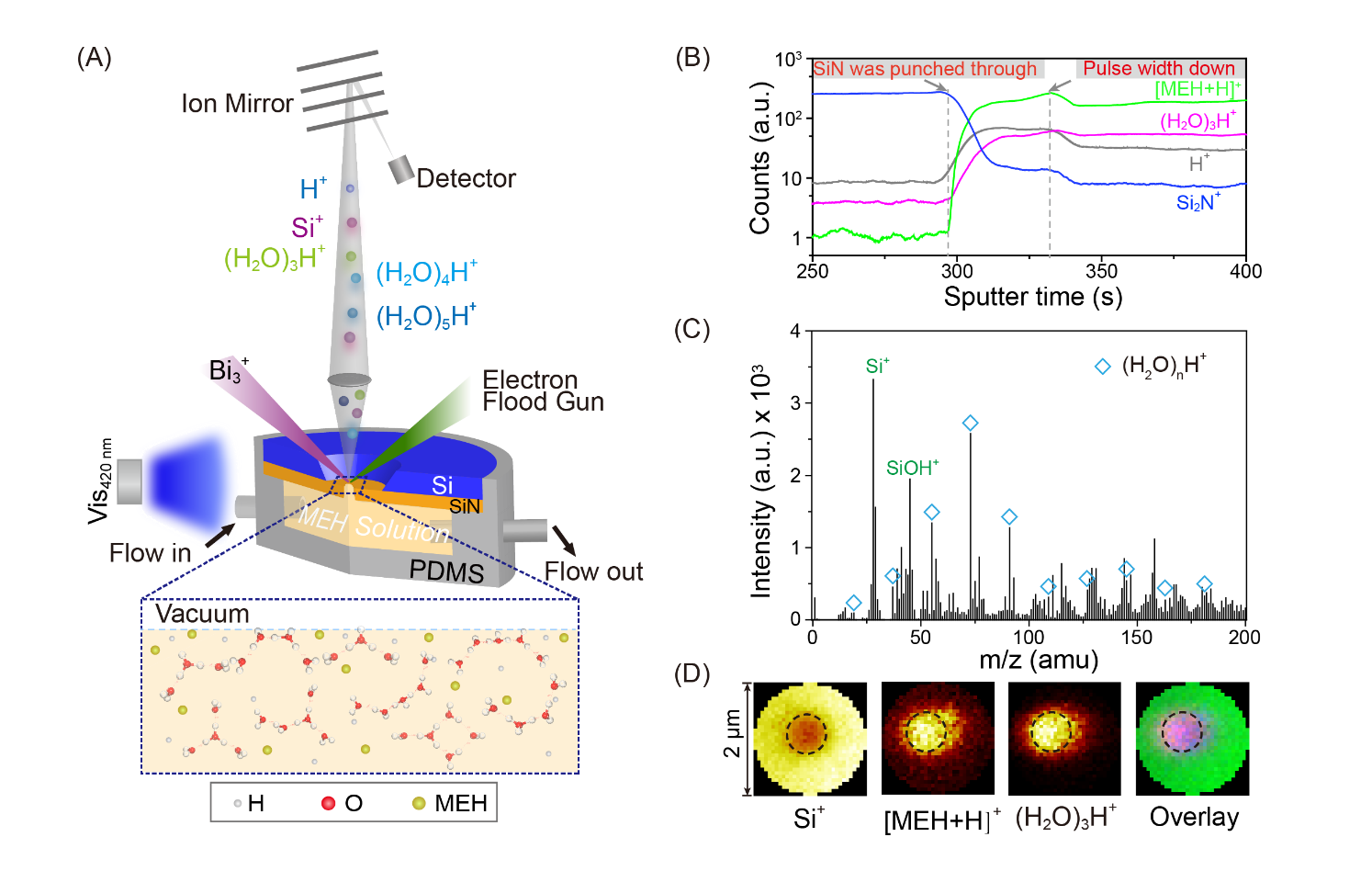


**Supplementary Figure 5.** (A) Schematic illustration of the hybrid light/ToF-SIMS technique; (B) The dynamic depth profiling by ToF-SIMS for 0.5 mM MEH solution in the positive mode. SiN membrane was punched through at around 295 s with the dramatically increased intensity of [MEH+H]^+^ and decreased signal of Si_2_N^+^; (C) Positive mass spectrum of the liquid-vacuum interface for 0.5 mM MEH solution in the microfluidic reactor, a series of blue rhombus represent the protonated water clusters (H_2_O)_n_H^+^; (D) The reconstructed 2D images of Si^+^, [MEH+H]^+^ and (H_2_O)_3_H^+^. The brighter area represents the higher intensity.


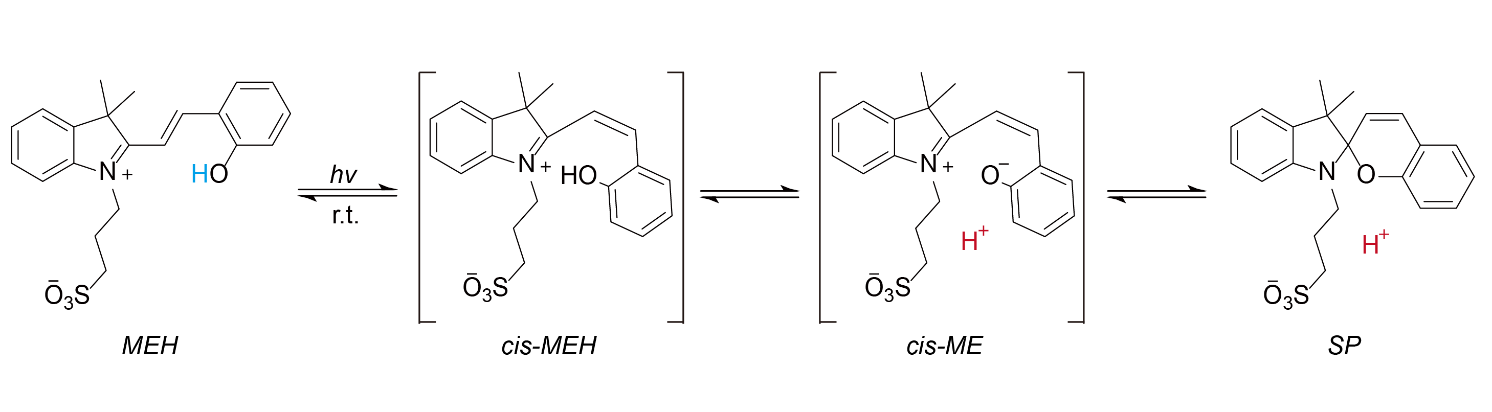


**Supplementary Figure 6.** Scheme of photochemical reaction process of MEH under visible light.


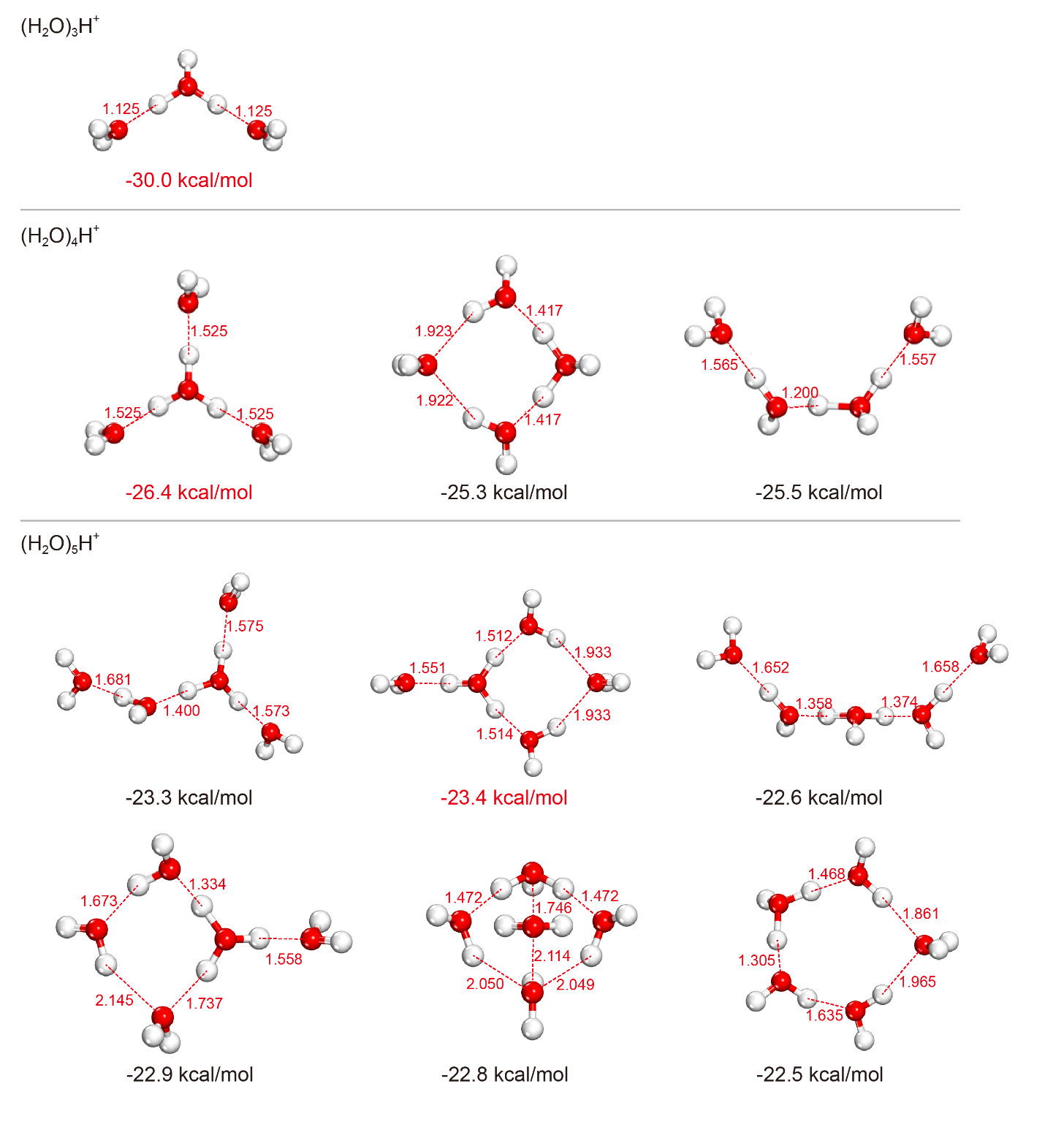


**Supplementary Figure 7.** Geometric isomers for (H_2_O)_n_H^+^ (n = 3, 4 and 5) clusters and the corresponding interaction energy (∆*E,* kcal/mol) between hydronium ion and waters for per water molecule were calculated in the theoretical level of M06-2x/aug-cc-pVTZ. The unit of length for hydrogen bonds is Angstrom (Å).


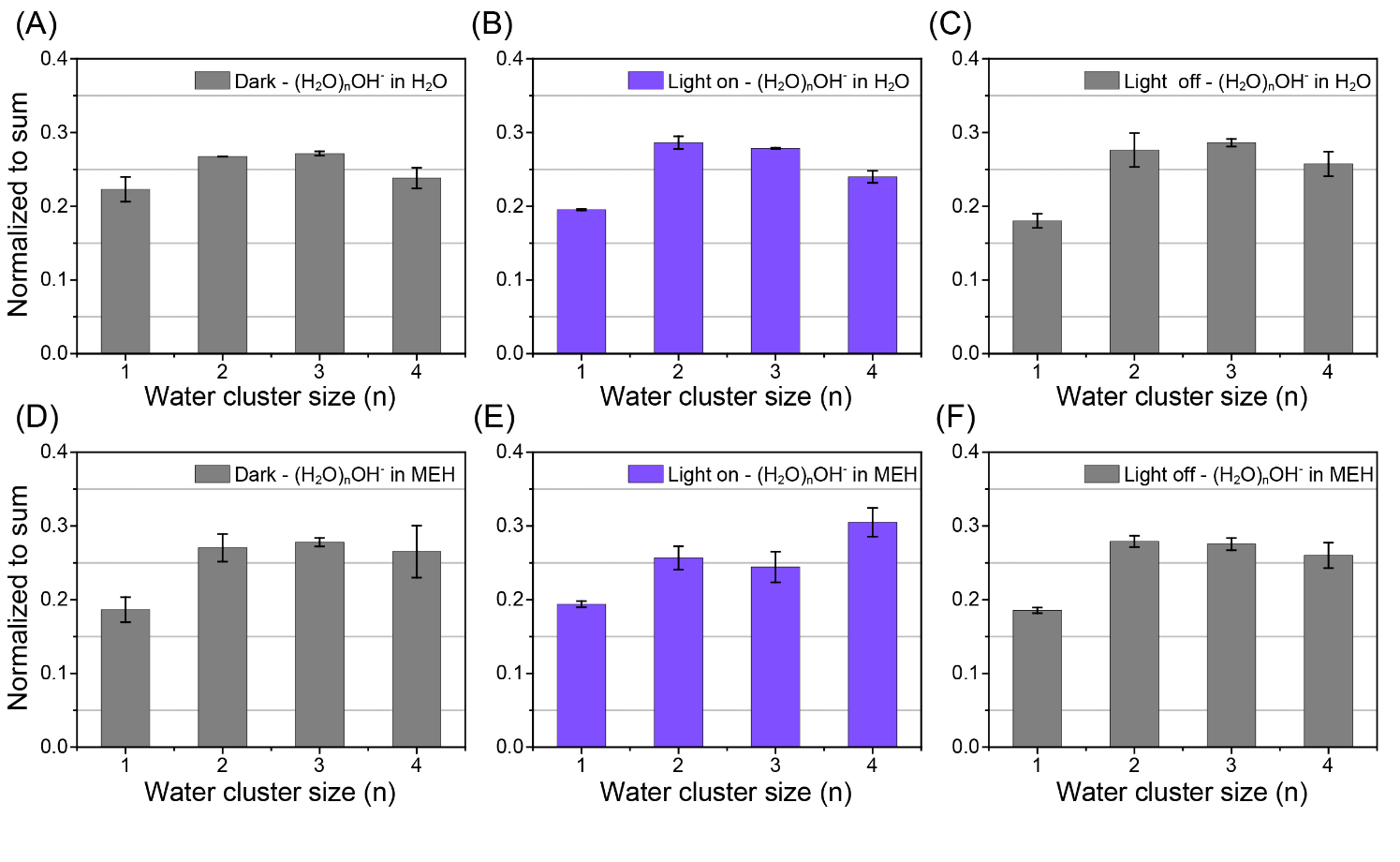


**Supplementary Figure 8.** Hydroxide water cluster size distributions in (A-C) pure water and (D-F) 0.5 mM MEH solutions in dark environment (A and D), under light illumination (B and E) and after light was turned off (C and F), respectively.


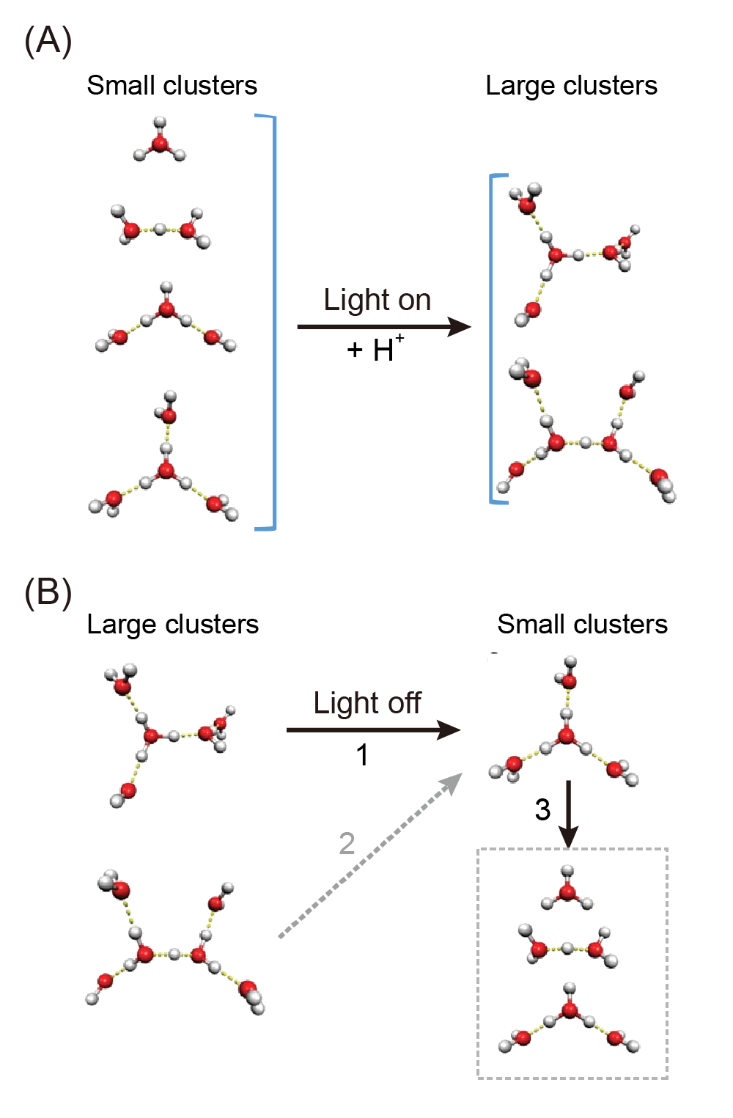


**Supplementary Figure 9.** The schematic diagram of water cluster structure changes in the MEH solution under dark (A) and illumination (B) condition.
